# Prevalence of Musculoskeletal Morbidity Among the Fishing Population of Pulicat, Tamil Nadu: A Community-Based Cross-Sectional Study

**DOI:** 10.7759/cureus.105702

**Published:** 2026-03-23

**Authors:** Stalin R, Ganesh Shanmugasundaram Anusuya, Gudluru Uday Sai

**Affiliations:** 1 Community Medicine, Sri Venkateswaraa Medical College Hospital and Research Institute, Sri Venkateswaraa University, Chennai, IND

**Keywords:** ergonomic, fishing community, nordic musculoskeletal questionnaire, occupational health hazard, work-related musculoskeletal morbidity

## Abstract

Introduction: Fishing is a physically demanding occupation involving repetitive movements, heavy lifting, prolonged standing, and unstable working conditions, predisposing workers to work-related musculoskeletal disorders (WMSDs). Community-based evidence from traditional fishing populations in Tamil Nadu is limited.

Methods: A community-based cross-sectional study was conducted among 125 fishermen aged ≥18 years engaged in fishing for at least one year. Participants were selected through simple random sampling. Data were collected using a pretested structured questionnaire, and musculoskeletal symptoms were assessed using the Standardized Nordic Musculoskeletal Questionnaire. Descriptive statistics were computed, and associations were analyzed using the chi-squared test with statistical significance set at p≤0.05.

Results: The majority of participants were male (76.8%) and aged ≥40 years (60%), with most having >10 years of fishing experience (80.8%) and working >8 hours per day (68.8%). Musculoskeletal pain was highly prevalent, particularly in the lower back (83.2%), shoulders (73.6%), and knees (70.4%) over the past 12 months. Longer working hours (>8 hours/day) and greater duration of fishing (>10 years) were significantly associated with increased prevalence of musculoskeletal pain, especially in the lower back and knees. Male participants also reported significantly higher prevalence of shoulder and lower back pain.

Conclusion: Musculoskeletal disorders are highly prevalent among fishermen in Pulicat, predominantly affecting the lower back, shoulders, and knees. Prolonged working hours and extended occupational exposure are key associated factors. These findings highlight the need for targeted ergonomic interventions, structured occupational health education, and inclusion of fisherfolk in occupational health and social protection programs to mitigate the burden of musculoskeletal morbidity and improve overall work capacity.

## Introduction

Work-related musculoskeletal disorders (WMSDs) are painful conditions that occur as a result of repetitive strain and cumulative injury to muscles, joints, tendons, nerves, and other soft tissues in the body. Musculoskeletal disorders are significant in that they affect the physical well-being and productivity of workers and result in considerable economic costs at both individual and societal levels [1].

Musculoskeletal disorders are among the top causes of long-term disability and morbidity worldwide. They are debilitating in nature and result in decreased functional ability, poor quality of life, and decreased work efficiency in affected individuals [2]. Musculoskeletal disorders are more common in low- and middle-income countries, where most workers are engaged in informal and labor-intensive occupations and lack access to healthcare services [3-5].

Fishing is identified as a high-risk occupation for the occurrence of musculoskeletal disorders owing to repetitive movements, heavy manual handling, prolonged postures, and exposure to unfavorable environmental conditions. Activities such as hauling fishing nets, heavy lifting, and prolonged standing and balancing on unstable fishing vessels impose continuous biomechanical stresses on fisherfolk, who are at risk of musculoskeletal disorders, especially in the lower back, shoulder, neck, and lower limb regions [6-8].

In India, the fisheries sector is a significant contributor to the workforce and the national economy, with a substantial part of the workforce engaged in traditional, non-mechanized fishing activities. However, occupational health issues faced by fisherfolk have received little research and attention. A review of the existing literature identified that there is little or no information available about the musculoskeletal morbidity among fisherfolk in Tamil Nadu, especially in the coastal fishing population around Pulicat.

Pulicat, a fishing village on the northern coastline of Tamil Nadu, India, harbors a substantial workforce of traditional fishermen, with the majority of the workforce engaged in physically demanding manual fishing activities. Despite the occupational hazards associated with this traditional fishing activity, there is little or no available data on the musculoskeletal health of this workforce. Therefore, the present study was conducted to assess the prevalence of musculoskeletal morbidity among fisherfolk in Pulicat, Tamil Nadu, to evaluate the distribution of musculoskeletal symptoms across different body regions, and to examine associations between musculoskeletal morbidity and sociodemographic and occupational factors.

## Materials and methods

This community-based cross-sectional study was carried out in Pulicat in the Tiruvallur district of Tamil Nadu, India. The study population comprised both male and female aged 18 years and above who were actively involved in fishing or fishing-related occupations for a minimum duration of 12 months. Individuals with congenital musculoskeletal deformities and a history of recent trauma or surgery in the past six months or those who did not consent to participate were excluded.

The sample size was calculated using the formula $\mathrm{n}=4\mathrm{pq}/\mathrm{d}^{2}$. Assuming a 92.4% prevalence of musculoskeletal morbidity with a 5% absolute precision, the minimum required sample size was estimated as 113 [9]. After incorporating a 10% allowance for non-response, the final sample size was calculated to be 125. The sampling frame was prepared using the fishermen's cooperative society registers and supplemented by local community records to ensure the comprehensive coverage of eligible participants in the study area.

From this defined sampling frame, study participants were selected using simple random sampling by the lottery method. Data collection was performed through face-to-face interviews by the principal investigator using a pretested structured questionnaire. The questionnaire was administered in the local language, Tamil, to ensure better comprehension among participants.

The Standardized Nordic Musculoskeletal Questionnaire was used to obtain responses regarding musculoskeletal morbidity that the fisherman had experienced during the last one year. The questionnaire is a validated and reliable tool for assessing musculoskeletal pain in each of the body parts [10]. It has a sensitivity ranging between 66% and 92% and a specificity between 71% and 88% [11]. The questionnaire has a content validity index of 87.2%, a Cronbach's alpha of 0.965-0.966, and a corrected item-total correlation value above 0.6 [12]. One response, whether "Yes" or "No", was required for every question depicting each body part separately.

Ethical approval was obtained from the Institutional Ethics Committee of Sri Venkateswaraa Medical College Hospital and Research Institute (IEC reference number: 001/12/2025/IEC/SVMCHRI) prior to the commencement of the study, and written informed consent was obtained from all participants. Collected data were entered into Microsoft Excel (Microsoft Corporation, Redmond, Washington, United States) and analyzed using IBM SPSS Statistics for Windows, Version 25.0 (IBM Corp., Armonk, New York, United States). Descriptive statistics were used to summarize the variables, and associations between musculoskeletal morbidity and selected sociodemographic and occupational factors were analyzed using the chi-squared test with statistical significance set at p≤0.05.

## Results

A total of 125 fishermen participated in the study. As shown in Table 1, the largest proportion belonged to the 40-49-year age group (38, 30.4%), followed by those aged ≥50 years (37, 29.6%). Males constituted 96 (76.8%) of the participants. Most were married (112, 89.6%). Educational attainment was generally low, with 39 (31.2%) being illiterate and 44 (35.2%) being educated up to the primary level. According to the Modified BG Prasad classification, the majority were in Class IV (44, 35.2%) and Class III (37, 29.6%). 

**Table 1:** Sociodemographic characteristics of the study participants (n=125)

Variable	Category	Frequency (n)	Percentage (%)
Age (years)	18-29	18	14.4
	30-39	32	25.6
	40-49	38	30.4
	≥50	37	29.6
Gender	Male	96	76.8
	Female	29	23.2
Marital status	Married	112	89.6
	Unmarried	13	10.4
Education	Illiterate	39	31.2
	Primary	44	35.2
	Secondary	26	20.8
	Higher secondary	9	7.2
	Diploma	4	3.2
	Graduate	3	2.4
Socioeconomic class (Modified BG Prasad)	Class I	6	4.8
	Class II	18	14.4
	Class III	37	29.6
	Class IV	44	35.2
	Class V	20	16

The occupational characteristics are detailed in Table 2. A large proportion had more than 10 years of fishing experience (101, 80.8%). Prolonged working hours were common, with 86 (68.8%) working more than eight hours daily. The most frequently reported tasks were net handling (91, 72.8%) and lifting heavy loads (74, 59.2%), followed by boat rowing/navigation (68, 54.4%) and fish sorting/cleaning (57, 45.6%).

**Table 2:** Occupational profile and work-related characteristics of the study participants (n=125) *Multiple responses were allowed; therefore, percentages may exceed 100%.

Variable	Category	Frequency (n)	Percentage (%)
Years of fishing experience	<10 years	24	19.2
	>10 years	101	80.8
Average working hours/day	≤8 hours	39	31.2
	>8 hours	86	68.8
Nature of work*	Net handling	91	72.8
	Boat rowing/navigation	68	54.4
	Fish sorting/cleaning	57	45.6
	Lifting heavy loads	74	59.2

The prevalence of musculoskeletal morbidity is presented in Table 3. During the preceding 12 months, lower back pain was the most frequently reported complaint (104, 83.2%), followed by shoulder pain (92, 73.6%), knee pain (88, 70.4%), and ankle/foot pain (73, 58.4%). Pain in the last seven days showed a comparable distribution, with lower back pain reported by 89 (71.2%), shoulder pain by 76 (60.8%), and knee pain by 74 (59.2%). Elbow and wrist/hand symptoms were less commonly reported.

**Table 3:** Prevalence of musculoskeletal morbidity among the study participants (n=125)

Body parts	Pain during the last 12 months n (%)	Pain during the last 7 days n (%)	No pain n (%)
Neck	38 (30.4)	27 (21.6)	87 (69.6)
Shoulder	92 (73.6)	76 (60.8)	33 (26.4)
Elbow	21 (16.8)	18 (14.4)	104 (83.2)
Wrists/hands	28 (22.4)	23 (18.4)	97 (77.6)
Upper back	44 (35.2)	36 (28.8)	81 (64.8)
Lower back	104 (83.2)	89 (71.2)	21 (16.8)
Thighs/hips/buttocks	33 (26.4)	27 (21.6)	92 (73.6)
Knees	88 (70.4)	74 (59.2)	37 (29.6)
Ankles/feet	73 (58.4)	63 (50.4)	52 (41.6)

The association between gender and musculoskeletal pain is shown in Table 4. A significantly higher proportion of males reported shoulder pain (75, 78.1%) compared to females (17, 58.6%) (p=0.03). Similarly, lower back pain was more frequent among males (84, 87.5%) than females (20, 69%) (p=0.01). No statistically significant association was observed between gender and pain in other body regions.

**Table 4:** Association between gender and musculoskeletal pain among fishermen (n=125) *p<0.05: significant (Pearson's chi-squared test)

Body parts	Status	Male n (%)	Female n (%)	Chi-square	P-value
Neck	No pain	66 (68.8)	21 (72.4)	0.14	0.71
	Pain	30 (31.2)	8 (27.6)		
Shoulder	No pain	21 (21.9)	12 (41.4)	4.36	0.03*
	Pain	75 (78.1)	17 (58.6)		
Elbow	No pain	80 (83.3)	24 (82.8)	0.01	0.94
	Pain	16 (16.7)	5 (17.2)		
Wrists/hands	No pain	73 (76)	24 (82.8)	0.58	0.45
	Pain	23 (24)	5 (17.2)		
Upper back	No pain	58 (60.4)	23 (79.3)	3.49	0.062
	Pain	38 (39.6)	6 (20.7)		
Lower back	No pain	12 (12.5)	9 (31)	5.47	0.01*
	Pain	84 (87.5)	20 (69)		
Thighs/hips/buttocks	No pain	68 (70.8)	24 (82.8)	1.63	0.20
	Pain	28 (29.2)	5 (17.2)		
Knees	No pain	26 (27.1)	11 (37.9)	1.26	0.26
	Pain	70 (72.9)	18 (62.1)		
Ankles/feet	No pain	38 (39.6)	14 (48.3)	0.69	0.41
	Pain	58 (60.4)	15 (51.7)		

As presented in Table 5, daily working hours demonstrated a significant relationship with musculoskeletal complaints. Participants working more than eight hours per day reported a higher prevalence of shoulder pain (68, 79.1%) (p=0.03), lower back pain (76, 88.4%) (p=0.02), and knee pain (67, 77.9%) (p<0.001). Associations for upper back and thigh/hip/buttock pain were of borderline significance, while elbow, wrist/hand, and ankle/foot pain were not significantly associated with working duration.

**Table 5:** Association between daily working hours and musculoskeletal pain among fishermen (n=125) *p<0.05: significant (Pearson's chi-squared test)

Body parts	Status	≤8 hours n (%)	>8 hours n (%)	Chi-square	P-value
Neck	No pain	16 (41)	71 (82.6)	21.87	0.00*
	Pain	23 (59)	15 (17.4)		
Shoulder	No pain	15 (38.5)	18 (20.9)	4.24	0.03*
	Pain	24 (61.5)	68 (79.1)		
Elbow	No pain	35 (89.7)	69 (80.2)	1.73	0.18
	Pain	4 (10.3)	17 (19.8)		
Wrists/hands	No pain	33 (84.6)	64 (74.4)	1.60	0.20
	Pain	6 (15.4)	22 (25.6)		
Upper back	No pain	30 (76.9)	51 (59.3)	3.65	0.05
	Pain	9 (23.1)	35 (40.7)		
Lower back	No pain	11 (28.2)	10 (11.6)	5.27	0.02*
	Pain	28 (71.8)	76 (88.4)		
Thighs/hips/buttocks	No pain	33 (84.6)	59 (68.6)	3.53	0.05
	Pain	6 (15.4)	27 (31.4)		
Knees	No pain	18 (46.2)	19 (22.1)	7.45	0.00*
	Pain	21 (53.8)	67 (77.9)		
Ankles/feet	No pain	20 (51.3)	32 (37.2)	2.18	0.13
	Pain	19 (48.7)	54 (62.8)		

The relationship between years of fishing experience and musculoskeletal pain is summarized in Table 6. Fishermen with more than 10 years of experience reported significantly higher lower back pain (88, 87.1%) compared to those with less experience (p=0.01). Knee pain was also more common among those with longer occupational exposure (76, 75.2%) (p=0.01). Although symptoms were generally more frequent in the experienced group across most anatomical regions, statistical significance was not observed for other body sites.

**Table 6:** Association between years of fishing experience and musculoskeletal pain among fishermen (n=125) *p<0.05: significant (Pearson's chi-squared test)

Body parts	Status	<10 years n (%)	>10 years n (%)	Chi-square	P-value
Neck	No pain	15 (62.5)	72 (71.3)	0.70	0.40
	Pain	9 (37.5)	29 (28.7)		
Shoulder	No pain	9 (37.5)	24 (23.8)	1.88	0.16
	Pain	15 (62.5)	77 (76.2)		
Elbow	No pain	22 (91.7)	82 (81.2)	1.52	0.21
	Pain	2 (8.3)	19 (18.8)		
Wrists/hands	No pain	21 (87.5)	76 (75.2)	1.67	0.19
	Pain	3 (12.5)	25 (24.8)		
Upper back	No pain	18 (75)	63 (62.4)	1.35	0.24
	Pain	6 (25)	38 (37.6)		
Lower back	No pain	8 (33.3)	13 (12.9)	5.80	0.01*
	Pain	16 (66.7)	88 (87.1)		
Thighs/hips/buttocks	No pain	19 (79.2)	73 (72.3)	0.47	0.49
	Pain	5 (20.8)	28 (27.7)		
Knees	No pain	12 (50)	25 (24.8)	5.93	0.01*
	Pain	12 (50)	76 (75.2)		
Ankles/feet	No pain	13 (54.2)	39 (38.6)	1.93	0.16
	Pain	11 (45.8)	62 (61.4)		

## Discussion

The present study has identified a high prevalence rate of musculoskeletal disorders among the fishermen population in Pulicat, thus emphasizing the high burden of health problems associated with fishing activities. The high rate of back pain, followed by shoulder, knee, and ankle or foot pain, is in accordance with the study findings on the musculoskeletal disorders observed in the fishing and fish processing population in various coastal areas of India and other countries [13,14]. The nature of the work, which involves frequent bending, lifting heavy objects, and standing, along with the exposure to unstable work environments, may lead to chronic pain, which is a major musculoskeletal disorder, because of the constant mechanical load on the musculoskeletal system [15].

The chronic nature of the musculoskeletal disorders, which is reflected by the high pain prevalence over the past 12 months and the recent seven-day period, thus points to the chronic nature of the musculoskeletal disorders observed in the fishermen population, which is in accordance with the study findings on the chronic nature of the musculoskeletal disorders observed in the fish processing population and the fisherwomen population, who are exposed to chronic stress with less recovery time [13,16]. The high rate of axial and lower limb pain observed in the study is in accordance with the study findings on the musculoskeletal disorders associated with the fishing activities, which may be attributed to the handling of the fishing nets and lifting heavy objects.

Gender-based analysis showed that there was a higher occurrence of shoulder pain and lower back pain in males. This may be attributed to task allocation in the fishing population. In the fishing population, men may be involved in tasks that require physical exertion, such as hauling the fishing nets, rowing the boats, and lifting fish from the nets. In contrast, women may be involved in tasks that require less physical exertion, such as fish processing. Studies have shown that women involved in fish processing have varying anatomical manifestations of musculoskeletal disorders. This suggests that task-specific exposure to musculoskeletal load rather than gender per se determines the occurrence of musculoskeletal disorders [16,17].

A significant relationship was observed between daily working hours and musculoskeletal pain in the neck, shoulder, lower back, and knee regions. Longer working hours contribute to fatigue because the muscles have less opportunity to rest. The relationship between working hours and musculoskeletal pain has been observed in the fish processing population in India as well as internationally.

Years of fishing experience were found to be significantly associated with increased incidence of low back and knee pain, which indicates the cumulative effect of occupational exposure. The chronic effect of repetitive loading, degenerative changes, and the effect of aging on musculoskeletal resilience and the increased susceptibility of the workforce with longer fishing experience can be attributed to the increased risk among the fishing workforce with longer fishing experience. Similar findings have been reported among experienced fishing workers and fisherwomen, where the effect of longer fishing experience was found to be one of the major determinants for musculoskeletal disorders [13,18].

Limitations

Although this study provides valuable community-based insights into musculoskeletal morbidity among fisherfolk, there are certain limitations. The use of self-reported data from the Standardized Nordic Musculoskeletal Questionnaire is subject to recall bias, particularly for symptoms over the past 12 months, and may also be influenced by social desirability bias and occupational stigma. The cross-sectional design precludes any causal inference. As the study was conducted in a single coastal community, the findings may have limited generalizability to other fishing populations. 

## Conclusions

This study demonstrates a high prevalence of musculoskeletal disorders among fishermen in Pulicat, with the lower back, shoulders, and knees being the most commonly affected regions. The persistence of these conditions reflects the continuous physical strain associated with fishing activities. These findings underscore the need for targeted preventive measures, including ergonomic modifications of fishing practices, provision of adequate rest periods, and health education on safe work techniques. At the policy level, integration of occupational health services for fisherfolk, periodic screening for musculoskeletal disorders, and inclusion under social security and welfare schemes are essential to reduce disability and improve overall health outcomes.
